# Clinical Determinants of Functional Independence at Discharge in Patients With Spinal Cord Dysfunction Due to Metastatic Spinal Tumors

**DOI:** 10.1016/j.arrct.2025.100549

**Published:** 2025-11-20

**Authors:** Hiroyuki Ase, Kiyomi Miura, Tsukasa Yoshida, Eriko Kitahara, Tatsuya Takagi, Kaoru Honaga, Akira Tanuma, Mami Tani, Yuhei Murakami, Aiko Ishikawa, Reina Isayama, Toshiyuki Fujiwara

**Affiliations:** a Department of Rehabilitation Medicine, Juntendo University Hospital, Bunkyo-ku, Tokyo, Japan; b Department of Rehabilitation Medicine, Juntendo University Graduate School of Medicine, Bunkyo-ku, Tokyo, Japan; c Department of Orthopedic Surgery, Juntendo University Hospital, Bunkyo-ku, Tokyo, Japan; d Palliative Care Center, Juntendo University Hospital, Bunkyo-ku, Tokyo, Japan; e Department of Rehabilitation Medicine, Juntendo Shizuoka Hospital, Bunkyo-ku, Tokyo, Japan; f Department of Physical Therapy, Juntendo University Faculty of Health Science, Bunkyo-ku, Tokyo, Japan

**Keywords:** Activities of daily living, Cancer rehabilitation, Metastatic spinal tumor, Prediction of functional independence, Rehabilitation, Spinal cord dysfunction

## Abstract

**Objectives:**

To identify the clinical factors associated with independence in activities of daily living (ADL) at discharge in patients with spinal cord dysfunction resulting from metastatic spinal tumors.

**Design:**

Retrospective cohort study.

**Setting:**

A single university hospital in Japan.

**Participants:**

A total of 153 patients (N=153) (median age 69 [interquartile range: 58.5-75.5]; 67% men) with spinal cord dysfunction caused by metastatic spinal tumors received inpatient rehabilitation during hospitalization between 2012 and 2022. The inclusion criteria were age ≥18 years, a diagnosis of spinal cord dysfunction resulting from metastatic bone tumors, and participation in inpatient rehabilitation.

**Interventions:**

Participants underwent standard inpatient rehabilitation care. No study-specific intervention was provided.

**Main Outcome Measures:**

Independence in ADL was defined as a Barthel index score of ≥85 at discharge. Data on demographics, clinical characteristics, and potential risk factors were extracted from medical records. Logistic regression analysis, adjusted for age, was conducted to identify predictors of independence in ADL at discharge.

**Results:**

Among the participants, 23 (15.0%) achieved independence in ADL at discharge. Logistic regression analysis identified the following 4 significant factors associated with reduced odds of independence in ADL: rapid-growing primary tumor type (odds ratio [OR]=5.93; 95% CI, 1.81-19.43), severe motor impairment at admission (OR=5.83; 95% CI, 1.89-18.00), elevated C-reactive protein/albumin ratio (OR=3.82; 95% CI, 1.53-17.80), and persistent movement-related pain (OR=1.34; 95% CI, 1.01-1.84).

**Conclusions:**

Tumor aggressiveness, neurologic severity, systemic inflammation, and persistent pain significantly influenced independence in ADL at discharge. Early identification of these risk factors may guide individualized rehabilitation planning and optimize functional outcomes in this population.

## Highlights

• Rapid cancer, severe paralysis, high C-reactive protein to albumin ratio, and persistent pain lower odds of independence in activities of daily living.
• Neurologic level and spinal instability cannot alone predict functional outcomes.
• Early recognition of these factors aids individualized rehabilitation and discharge planning.

Bone metastases are a frequent and severe complication in patients with advanced malignancies, particularly in those with breast, prostate, and lung cancers.^1^ Because of improvements in oncologic treatments, the number of patients living with bone metastases has increased globally.^2^ Patients with bone metastases often experience a substantial decline in quality of life because of pain, reduced mobility, and skeletal-related events such as pathologic fractures or spinal cord compression.^3^ When bone metastases involve the spine, they may lead to metastatic spinal cord compression or spinal cord dysfunction resulting from tumor-induced instability or collapse of the vertebrae. These events can lead to nontraumatic spinal cord injury (SCI), which is associated with sudden motor and sensory impairments. Compared with traumatic SCI, patients with bone metastases often have limited life expectancy, which restricts the time available for functional recovery and the re-establishment of daily activities.

Early rehabilitation is recommended for patients with bone metastases to prevent further decline in function and facilitate discharge home.^4^ However, clinical decision making in this population is complex because of the heterogeneity in tumor types, neurologic involvement, and overall prognosis. Although prognostic tools such as the Katagiri,^5^ Tokuhashi,^6^ or Tomita^7^ scoring systems are available to estimate survival, limited evidence has been published regarding how to predict functional outcomes, particularly the likelihood of achieving independence in activities of daily living (ADL) at discharge. Previous studies have examined predictors of ambulatory independence in patients with SCI caused by spinal metastases,^8^ as well as postoperative functional prognosis in patients with spinal metastases.^9^, ^10^, ^11^ However, these studies have focused mainly on neurologic recovery or survival time. Few studies have systematically examined predictors of independence in ADL among patients with spinal cord dysfunction caused by metastatic spinal tumors. This knowledge gap presents a significant challenge to rehabilitation professionals in selecting appropriate therapeutic strategies. Under these circumstances, accurate prediction of functional outcomes is essential for tailoring rehabilitation strategies—whether restorative, biomechanical, or compensatory. Clarifying these factors may assist clinicians in setting realistic rehabilitation goals and optimizing care strategies in a clinical context. On this basis, this study aimed to identify the clinical factors associated with independence in ADL at discharge among patients with spinal cord dysfunction resulting from metastatic spinal tumors.

## Methods

### Study design and participants

This retrospective cohort study was conducted at a single university hospital in Japan from January 2012 to December 2022. All data were anonymized before analysis. Follow-up data were collected from medical records, covering the entire hospitalization period until discharge. This study was reported following the Strengthening the Reporting of Observational Studies in Epidemiology statement.^12^ The inclusion criteria were (1) age ≥18 years; (2) diagnosed with spinal cord dysfunction because of metastatic bone tumors; (3) received inpatient rehabilitation; and (4) underwent assessments of ADL, motor function, and imaging evaluation. The exclusion criteria were (1) inability to assess ADL or motor function at the start of rehabilitation because of clinical limitations; (2) deterioration of physical function to an Eastern Cooperative Oncology Group Performance Status score ≤3 because of noncancer-related comorbidities before or after the initiation of rehabilitation; and (3) determination by the principal investigator that the patient was not suitable for inclusion in the study. This study was performed in accordance with the Declaration of Helsinki and approved by the Research Ethics Committee, Faculty of Medicine, Juntendo University (approval number: E23-0131). Given the retrospective nature of the study, the requirement for informed consent was waived, and an opt-out procedure was implemented.

### Data collection

Data were retrospectively collected from medical records. The extracted variables included patient demographics, length of hospital stay, and discharge destination. Independence in ADL was defined as a Barthel index (BI) score of ≥85 at discharge, consistent with previous studies that used this threshold to indicate functional independence.^13^^,^^14^ Patients were categorized into an ADL-independent group (BI≥85) or an ADL-dependent group (BI<85). Several potential risk factors for independence in ADL were examined, including the primary tumor type (classified according to Katagiri growth categories), neurologic level of injury, severity of motor impairment at rehabilitation initiation, the Spinal Instability Neoplastic Score (SINS),^15^ presence of movement-related pain, and the C-reactive protein to albumin ratio (CRP/Alb ratio) at rehabilitation initiation.

The primary cancer site is frequently used as a prognostic factor in survival prediction models for patients with bone metastases. Representative studies include those by Katagiri et al,^5^ Tokuhashi et al,^6^ and Tomita et al.^7^ Furthermore, Hosono et al^16^ reported that the histologic type of the primary tumor was the strongest prognostic factor, followed by preoperative paresis and pain in patients with spinal metastasis. In this study, we classified the primary cancer site into 3 categories based on the classification proposed by Katagiri et al^5^: slow growth (eg, hormone-dependent breast and prostate cancer, thyroid cancer, multiple myeloma, malignant lymphoma), moderate growth (eg, lung cancer treated with molecularly targeted drugs, hormone-independent breast and prostate cancer, renal cell carcinoma, endometrial and ovarian cancer, sarcoma), and rapid growth (eg, lung cancer without molecularly targeted drugs, gastrointestinal cancers, head and neck cancer, other urological cancers, melanoma, hepatocellular carcinoma, gall bladder cancer, cervical cancer, cancers of unknown origin).

The neurologic level of injury is also recognized as an important prognostic factor for functional outcomes. Higher-level lesions, particularly with cervical involvement, are generally associated with greater functional impairment and a lower probability of regaining independence in ADL compared with thoracic or lumbar lesions. Clinical guidelines and prior studies of SCI have consistently shown that functional outcomes, including mobility and self-care, vary considerably by neurologic level, with cervical lesions generally associated with poorer outcomes.^17^ In this study, the cervical level served as the reference category for the neurologic level.

The severity of motor impairment at the initiation of treatment is often used as a prognostic factor for functional outcomes in patients with spinal metastasis. In patients with cancer accompanied by spinal cord compression, those classified as Frankel A or B before treatment generally have a poor prognosis. By contrast, patients with Frankel grade C or higher are more likely to regain ambulation.^18^^,^^19^ Furthermore, the ability to walk before radiotherapy has been associated with better functional outcomes.^20^ Among the methods for assessing the severity of motor impairment, the American Spinal Injury Association (ASIA) Impairment Scale (AIS) is often utilized. The AIS has been reported to be superior to the Frankel classification in terms of diagnostic stability, demonstrating a lower rate of apparent neurologic deterioration regression from an incomplete to a complete status when classifying traumatic SCIs compared with the Frankel system.^21^ In addition, the AIS has been shown to have high inter-rater reliability, with experienced evaluators showing very high agreement for the AIS grade classification.^22^ Regarding construct validity, research focusing on the ASIA Motor Scale, a component of the AIS classification system, has suggested that using separate upper and lower extremity scales may provide a more accurate representation of motor function than a single total score.^23^ Although many previous studies on metastatic spinal cord compression have used the Frankel classification to describe neurologic status, this study employed the AIS because of its superior reliability and diagnostic stability.

The SINS is a scoring system used to assess spinal instability. A score of 0-6 indicates stability, 7-12 potential instability, and 13-18 instability. Greater instability suggests a stronger recommendation for surgical stabilization.^24^ In this study, we used the SINS score assessed at the initial diagnosis by an orthopedic oncologist.

Pain has also been reported as a factor hindering physical activity.^17^ Bone metastasis-related pain is typically managed with a combination of rest, pharmacologic therapy, radiation therapy, and interventional radiology. It has been reported that radiation therapy achieves ≥50% pain relief on average within 13 days.^25^ In this study, pain was evaluated using a numeric rating scale (NRS) at 3 weeks after the implementation of such combined treatment.

The CRP/Alb ratio, an indicator of systemic nutritional and inflammatory status, has been widely studied as a prognostic marker in various malignancies.^26^, ^27^, ^28^ Although few studies have directly demonstrated its association with functional outcomes in patients with cancer, some have reported that an elevated CRP/Alb ratio is related to poorer survival and, by implication, a decline in the prediction of functional outcomes.^29^^,^^30^ Given that a higher CRP/Alb ratio may reflect systemic deterioration that can lead to impaired physical activity, in this study, we adopted the CRP/Alb ratio as a potential risk factor for predicting functional outcomes. Laboratory data, including CRP and Alb, were obtained at rehabilitation initiation and before any surgical or oncologic treatment, thereby avoiding transient postoperative changes in CRP values.

### Statistical analysis

Descriptive statistics on patient characteristics and admission characteristics are presented as medians, percentages, and interquartile ranges (IQR). The chi-square test or the Mann–Whitney *U* test was used to test comparisons between the 2 groups. Patients were dichotomized based on their BI score at discharge, and comparisons were made between the ADL-independent and ADL-dependent groups to identify the factors associated with independence in ADL. In addition to reporting odds ratios (ORs) and 95% CIs, effect sizes (Cliff’s delta for continuous or ordinal variables and Cramer’s V for categorical variables) were calculated to complement significance testing, given the exploratory design of this study and limited sample size. The magnitudes of Cliff’s delta were interpreted according to established thresholds (|δ|=0.147 small, 0.33 medium, 0.474 large),^31^ and those of Cramer’s V were interpreted as V=0.1 small, 0.3 medium, and 0.5 large.^32^

Logistic regression was performed to examine potential predictors of ADL outcomes. To avoid model overfitting caused by the small number of ADL-independent cases, analyses were limited to univariate models with adjustment for age only. Age was selected a priori as a covariate based on its clinical relevance and known association with functional outcomes. We modeled the odds of ADL dependence (BI<85); therefore, an OR>1 indicates increased odds of remaining ADL-dependent. The ORs with 95% CIs were reported as measures of effect size, and their magnitudes were interpreted according to established benchmarks (OR=1.5 small, 2.5 medium, 4.3 large).^33^ The level of statistical significance was set at *P*<.05. All analyses were conducted using JMP Pro 18.1.^a^

Given the retrospective nature of this study and the limited number of eligible cases, priori sample size calculation was not performed. The sample size was determined by the number of patients who met the inclusion criteria during the study period. This study is intended to serve as an exploratory analysis to identify potential factors associated with independence in ADL at discharge. Given the exploratory purpose and limited number of ADL-independent cases, no sensitivity analyses were performed.

## Results

**Fig 1:**
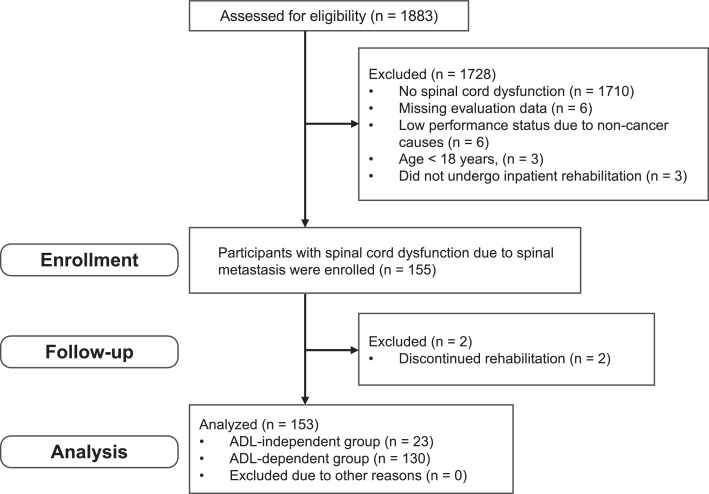
Flow diagram of patient selection. “Enrollment” refers to patients with spinal cord dysfunction caused by spinal metastasis who met all the inclusion criteria, did not meet any exclusion criteria, and initiated inpatient rehabilitation. “Follow-up” refers to the inpatient period until discharge, during which time, outcome data were retrospectively collected from medical records. Of the 1883 individuals screened, 155 were enrolled. During follow-up, 2 patients were excluded because they had discontinued rehabilitation, resulting in a final analytical cohort of 153 patients.

The participant flow diagram following the Strengthening the Reporting of Observational Studies in Epidemiology statement is shown in figure 1. Among the 1883 patients diagnosed with bone metastases during the study period, 1728 were excluded for the reasons including absence of spinal cord dysfunction (n=1710), missing evaluation data (n=6), low performance status caused by noncancer causes (n=6), age <18 years (n=3), and no inpatient rehabilitation (n=3). As a result, a total of 155 patients were therefore included in the study. During follow-up, 2 patients discontinued rehabilitation, leaving 153 patients for the final analysis.

### Patient characteristics

A total of 153 patients with spinal cord dysfunction caused by metastatic spinal tumors were included in this study (table 1). There were no missing data in the variables used for the analysis. The median age was 69 (IQR, 58.5-75.5) years, and the cohort consisted of 102 men (67%) and 51 women (33%). The median length of hospital stay was 47.0 days (IQR, 31.0-64.0), and the discharge destinations were as follows: home (48%), care facility (24%), transfer to another hospital (5%), and in-hospital death (23%).

**Table 1:** Patient characteristics.

Patient Background and Clinical Characteristics on Admission		Total (n=153)	ADL-Dependent Group (n=130)	ADL-Independent Group (n=23)	*P* Value	Effect Size	Interpretation
Patient characteristics							
Sex	Male/female	102 (67%)/51 (33%)	86 (66%)/44 (34%)	16 (70%)/7 (30%)	.749	Cramer’s V=0.026	Negligible
Age	Median (IQR)	69 (58.5-75.5)	69 (60.8-77.0)	63 (50.0-71.0)	.032	Cliff’s delta=0.286	Small
Primary cancer site		Lung: 34 (22%), Prostate: 21 (14%), Breast: 15 (10%), Liver: 13 (8%), Colon: 12 (8%), Hematologic: 9 (6%), Others: 49 (32%)	Lung: 28 (22%), Prostate: 16 (12%), Breast: 11 (8%), Liver: 12 (9%), Colon: 12 (9%), Hematologic: 4 (3%), Others: 47 (37%)	Lung: 6 (26%), Prostate: 5 (22%), Breast: 4 (17%), Liver: 1 (4%), Colon: 0 (0%), Hematologic: 5 (22%), Others: 2 (9%)	.001	Cramer’s V=0.378	Medium
Neurologic level of injury	C/T/L/S	11(7%)/109(71%)/ 27(18%)/6(4%)	9 (7%)/92 (71%)/25 (19%)/4 (3%)	2 (9%)/17 (74%)/2 (9%)/2 (9%)	.405	Cramer’s V=0.138	Small
Katagiri score	0-3/4-6/7-10	15 (12%)/40 (30%)/78 (58%)	10 (8%)/36 (28%)/84 (65%)	6 (17%)/10 (43%)/7 (30%)	.004	Cramer’s V=0.266	Small
SINS	Stable/intermediate/unstable	18 (12%)/90 (59%)/45 (29%)	14 (11%)/79 (61%)/37 (28%)	4 (30%)/12 (52%)/7 (30%)	.614	Cramer’s V=0.080	Negligible
Surgery	No/Yes	129 (84%)/24 (16%)	114 (88%)/16 (12%)	15 (65%)/8 (35%)	.006	Cramer’s V=0.221	Small
Radiation therapy	No/Yes	10 (7%)/143 (93%)	7 (5%)/123 (95%)	3 (13%)/20 (87%)	.170	Cramer’s V=0.111	Small
Length of hospital stay	Median (IQR)	47.0 (31.0-64.0)	50.5 (32.8-66.3)	41.0 (29.0-51.0)	.029	Cliff’s delta=0.002	Negligible
Discharge destination	Home/facility/transfer/death	73 (48%)/37 (24%)/8 (5%)/35 (23%)	53 (41%)/36 (28%)/6 (5%)/35 (27%)	21 (91%)/0 (0%)/2 (9%)/0 (0%)	<.001	Cramer’s V=0.363	Medium
Characteristics upon admission							
ECOG PS	0/1/2/3/4	0 (0%)/6 (4%)/13 (8%)/47 (30%)/87 (58%)	0 (0%)/2 (2%)/9 (7%)/38 (29%)/81 (62%)	0 (0%)/4 (17%)/4 (17%)/8 (35%)/7 (30%)	<.001	Cramer’s V=0.350	Medium
Walking ability (FAC)	0-2/3-5	133 (86%)/20 (13%)	117 (90%)/13 (10%)	16 (70%)/7 (30%)	.007	Cramer’s V=0.217	Small
AIS before treatment	A/B/C/D	8 (5%)/13 (8%)/70 (46%)/62 (41%)	8 (6%)/13 (10%)/63 (48%)/46 (35%)	0 (0%)/0 (0%)/7 (30%)/16 (70%)	.013	Cramer’s V=0.265	Small
Barthel Index	Median (IQR)	20 (5-50)	15 (5-40)	55 (35-70)	<.001	Cliff’s delta=−0.581	Large
Pain at admission	No/Yes	80 (52%)/73 (48%)	69 (54%)/61 (46%)	11 (48%)/12 (52%)	.642	Cramer’s V=0.038	Negligible
BBD	No/Yes	95 (62%)/58 (38%)	77 (59%)/53 (41%)	18 (78%)/5 (22%)	.083	Cramer’s V=0.140	Small
Albumin	Median (IQR)	3.3 (2.9-3.8)	3.3 (2.9-3.7)	3.9 (3.5-4.3)	<.001	Cliff’s delta=−0.464	Medium
CRP	Median (IQR)	1.5 (0.4-6.8)	2.1 (0.5-7.7)	0.5 (0.2-2.4)	.005	Cliff’s delta=0.683	Medium

### Comparison of ADL-independent and ADL-dependent groups

At the time of discharge, 23 patients (15%) were ADL-independent (BI≥85), whereas the remaining 130 patients (85%) were ADL-dependent. The characteristics of the 2 groups are shown in table 1. Compared with patients in the ADL-dependent group, those in the ADL-independent group had a significantly shorter median [IQR] hospital stay (41.0 [29.0-51.0] days vs 50.5 [32.8-66.3] days, *P*=.029) and a markedly higher rate of discharge (91% vs 41%, *P*<.001; Cramer’s V=0.363; medium effect size). Significant differences were observed in age (*P*=.032; Cliff’s δ=−0.286; small effect size), primary cancer site (*P*=.001; Cramer’s V=0.378; medium effect size), Katagiri score (*P*=.004; Cramer’s V=0.266; a small effect size), surgery (*P*=.006; Cramer’s V=0.221; small effect size), and several admission characteristics, including Eastern Cooperative Oncology Group Performance Status, AIS, BI, and CRP (medium to large effect sizes). By contrast, no significant differences (with negligible or small effect sizes) in sex distribution, SINS, or bladder and bowel dysfunction were found.

In table 1, differences in several patient characteristics were found between groups; however, not all of these were considered explanatory variables in the regression analysis. Explanatory variables were selected based on clinical relevance and the previous literature, and these were subsequently examined in univariate logistic regression to identify independent predictors of dependence in ADL (table 2).

**Table 2:** Logistic regression analysis of factors affecting dependence in activities of daily living at discharge.

Variable	Univariate		Univariate (Adjusted for Age)		
	OR (95% CI)	*P* Value	OR (95% CI)	*P* Value	Effect Size
Sex					
Male	0.86 (0.33-2.23)	.749			
Age					
Per 1-y increase	1.04 (1.01-1.08)	.019*			
Primary tumor type†					
Slow	1.00 (reference)				
Medium	3.73 (0.89-25.68)	.073	3.41 (0.57-20.48)	.180*	
Rapid	5.60 (2.12-16.04)	.001*	5.93 (1.81-19.43)	.003*	Large
Neurologic level of injury					
Cervical	1.00 (reference)				
Thoracic	1.20 (0.24-6.06)	.823			
Lumbar	2.78 (0.34-22.75)	.341			
Sacral	0.44 (0.05-4.37)	.487			
Severity of motor impairment at treatment start‡					
AIS<D	4.17 (1.66-11.54)	.002*	5.83 (1.89-18.00)	.002*	Large
SINS§					
Stable	1.00 (reference)				
Intermediate	1.89 (0.52-6.84)	.333			
Unstable	1.52 (0.38-6.16)	.554			
NRSǁ at 3 wk					
Per 1-point increase	1.35 (1.03-1.77)	.028*	1.34 (1.01-1.84)	.048*	Negligible
Inflammation marker¶					
CRP/Alb ratio	6.52 (2.34-31.38)	.004*	3.82 (1.53-17.80)	<.001*	Medium

⁎ *P*<.05.

† The primary tumor type was categorized into 3 groups–slow, medium, and rapid–based on the prognostic scoring system developed by Katagiri et al.^5^

‡ The degree of incomplete paralysis was classified into 2 groups (D and ≤C) according to the ASIA Impairment Scale.

§ SINS: on the basis of the Spinal Instability Neoplastic Score, patients were categorized into 3 groups: stable, intermediate, and unstable.

ǁ Movement-related pain at 3 wk after the start of treatment was assessed using the NRS.

¶ As an inflammation marker, the CRP/albumin ratio at the start of treatment was calculated.

### Logistic regression analysis of predictors of dependence in ADL

A logistic regression analysis was conducted using age as a covariate to identify clinical predictors of dependence in ADL. Because of the small number of outcome events, including more variables in the regression model risked overfitting. The results are summarized in table 2.

The type of primary cancer, classified according to the Katagiri scoring system into slow, moderate, or rapid growth categories, was significantly associated with ADL outcomes. Patients with cancers classified as rapid growth type had higher odds of ADL dependence (OR=5.93; 95% CI, 1.81-19.43, *P*=.003; large effect size). In terms of neurologic function, patients with more severe motor impairment at rehabilitation initiation, categorized using the ASIA Impairment Scale (AIS C or lower vs AIS D), also had higher odds of ADL dependence (OR=5.83; 95% CI, 1.89-18.00; *P*=.002; large effect size). This finding underscores the impact of initial neurologic status on functional outcomes, even in patients with spinal metastases, who tend to have shorter rehabilitation periods. Movement-related pain was evaluated using the NRS at 3 weeks after the start of treatment. Persistent movement-related pain was significantly associated with ADL outcomes (OR=1.34; 95% CI, 1.01-1.84; *P*=.048), although the observed effect size was relatively small. Systemic inflammatory status, represented by the CRP/Alb ratio at the beginning of rehabilitation, was another strong predictor. A higher CRP/Alb ratio, which has been associated with a poor cancer prognosis, was associated with increased odds of ADL dependence (OR=3.82; 95% CI, 1.53-17.80; *P*<.001; medium effect size). This suggests that poor nutritional and inflammatory profiles at admission may reduce the potential benefit of rehabilitation.

These findings indicate that rapid tumor growth, severe motor impairment, elevated systemic inflammation, and persistent pain are clinical factors that significantly increase the odds of dependence in ADL, and thus effectively serve as factors that limit functional recovery in this population. However, in this study, neither the neurologic level of injury nor the SINS significantly impacted dependence in ADL at discharge.

## Discussion

This study identified key clinical factors that increase the risk of ADL dependence at discharge in patients with spinal cord dysfunction caused by metastatic spinal tumors. Logistic regression was used to model ADL dependence, but the results were interpreted as factors hindering independence in ADL. Logistic regression analysis revealed that rapid-growing primary cancers, severe motor impairment at rehabilitation initiation, an elevated CRP/Alb ratio, and persistent movement-related pain were significantly associated with a lower likelihood of independence in ADL. Although prognostic prediction after surgery for bone metastases and gait prediction for spinal metastases caused by bone metastases have been performed in previous studies, to the best of our knowledge, this is the first report detailing the prediction of functional outcomes for independence in ADL among patients with spinal dysfunction because of bone metastases.

In this study, independence in ADL was defined as a BI score of ≥85 at discharge. Although no universal cutoff for defining independence in ADL among patients with cancer has been established, several studies support the use of this threshold. For example, a survey regarding cerebrovascular disease found that a modified Rankin Scale score of 2—corresponding to the ability to perform all individual ADL without assistance—was associated with a BI cutoff score of 90.^34^ In the field of oncology, studies involving patients with hepatocellular carcinoma undergoing hepatectomy have defined BI scores <85 as indicating functional impairment or frailty, both of which are associated with worse surgical outcomes.^13^^,^^14^ Furthermore, the BI includes the items stair climbing (10 points) and getting into the bathtub (5 points), which may not be essential for home-based independent living depending on lifestyles and living environments. Considering these contextual factors and prior evidence, adopting a BI cutoff score of ≥85 is considered to provide a pragmatic and clinically meaningful definition of independence in ADL in the study population investigated in this study.

### Neurologic and systemic factors

The present findings align with earlier reports suggesting that neurologic status and systemic condition are significant determinants of functional outcomes in patients with spinal metastases.^35^ In this study, severe motor impairment at baseline, particularly AIS grade C or lower, was associated with higher odds of dependence in ADL (large effect size), underscoring the critical role of neurologic status. Similarly, systemic inflammation and malnutrition, reflected by a high CRP/Alb ratio, have been linked to impaired functional recovery and overall poor prognosis in oncology populations.^36^ The present findings confirmed this association with a medium effect size, reinforcing the prognostic utility of inflammatory and nutritional markers. Furthermore, the type of primary cancer, classified according to growth rate, was a strong predictor of ADL outcomes. Rapid growth categories of primary cancer were positively associated with dependence in ADL (large effect size). Slow-growing cancers (eg, breast or prostate) generally allow for longer extended rehabilitation periods and functional gains compared with rapidly progressing tumors. As cancer progresses, patients often develop cachexia and malnutrition, leading to a marked decline in exercise tolerance.^37^^,^^38^ This factor likely reflects biological aggressiveness and the feasibility of sustained rehabilitation interventions.

### Role of pain in functional outcomes

The findings of this study suggest that persistent pain is a significant factor increasing the risk of dependence in ADL. Ideally, independence in ADL could be predicted based on pain status at admission. However, in this study, approximately 50% of the patients in the ADL-independent and ADL-dependent groups were found to have reported experiencing pain at admission. Furthermore, because pain relief is a primary goal of treatment for bone metastases, predicting functional outcomes based solely on pain at admission was considered difficult. Therefore, pain was assessed using the NRS at 3 weeks after treatment initiation to reflect persistent pain. Although the statistical association remained significant, the effect size was relatively small, suggesting that the overall clinical impact of persistent pain on ADL outcomes may be modest. Nevertheless, persistent pain interfering with movement is clinically significant, as it may induce kinesiophobia and reduce participation in rehabilitation. In patients with spinal cord dysfunction because of metastatic spinal tumors, pain often tends to persist because of local instability or neural compression at the metastatic site.^39^ Persistent pain may lead to pain-related kinesiophobia, which can result in reduced physical activity and decreased engagement in rehabilitation.^40^ Accordingly, adequate pain control is a central component of supportive care for patients with metastatic spinal cord disease and is an essential factor in improving function.

### Impact of spinal instability and surgery on functional outcomes

Interestingly, as assessed by the SINS, vertebral instability did not emerge as a significant factor in this analysis. One possible explanation for this is that many patients with unstable lesions had already undergone surgical or radiological stabilization before rehabilitation initiation, thereby reducing its direct impact on ADL. On the other hand, some patients with unstable lesions did not receive surgical stabilization, which may have influenced the findings. Table 1 also indicates that the number of patients who underwent surgical intervention was smaller than the number of those assessed as having spinal instability. This suggests that vertebral instability does not necessarily equate to the decision to perform surgery. The treatment plan for bone metastases is also determined based on multifaceted considerations such as the patient’s general condition and future treatment plan. Therefore, it is clear that neither spinal instability nor the performance of surgery predicts functional outcomes. In rehabilitation medicine, it is essential to consider intervention programs in line with the treatment plan; however, the prediction of functional outcomes should be addressed independently.

### Implications for rehabilitation planning

The median length of hospital stay among our cohort was approximately 47 days, with about half of the patients discharged home and over 70% returning to a care setting, including facilities. This highlights the need for timely and targeted rehabilitation planning. In addition, the relatively high proportion of in-hospital deaths observed in our cohort reflects the inclusion of patients for whom intensive functional recovery was no longer realistic and who transitioned to palliative or supportive care after oncologic treatment. These findings highlight the importance of integrating prognostic assessment into rehabilitation planning. Biomarkers such as the CRP/Alb ratio, which are associated with poor survival in various malignancies, may help identify patients with limited life expectancy. In such cases, rehabilitation goals should shift promptly from intensive functional training to supportive approaches that focus on quality of life.

In contrast to patients with traumatic SCI, who typically undergo prolonged inpatient rehabilitation, individuals with metastatic spinal disease often require early reintegration into daily life. The present findings also emphasize the importance of evaluating not only the neurologic level of injury and injury severity, but also broader factors such as systemic inflammation, tumor biology, and pain persistence. By considering these elements early in the rehabilitation course, clinicians may better anticipate patient needs and implement individualized strategies, including compensatory techniques or environmental modifications, to support functional independence. Therefore, unlike patients with traumatic SCI, those with SCI caused by bone metastases should be approached with a multifaceted strategy aimed at achieving goals within a significantly shorter time frame, taking life expectancy, predicted functional outcomes, and treatment strategies into account.

Moreover, the interpretation of hospital length of stay should be considered in a health system context. In Japan, inpatient stays, particularly for those with spinal cord dysfunction caused by spinal metastases, often encompass both acute oncologic treatment and rehabilitation, which differs from settings where patients transfer to rehabilitation units. The reported durations of postmetastatic spinal cord compression rehabilitation vary widely, from 15 to 111 days.^35^ Thus, our median stay of 47 days may partly reflect system-specific practices rather than unusually prolonged hospitalization. In addition, cultural and systemic differences, such as the availability of family or community-based support, may further influence discharge timing.

### Study limitations

This study has several limitations. First, although the study period spanned 10 years, the number of ADL-independent patients was relatively small (n=23), which limited the statistical power and restricted the multivariate modeling. Only age was used as a covariate in the regression analysis, and the possibility of residual confounding cannot be excluded. Second, this was a single-center study conducted in Japan. Variability in treatment protocols, rehabilitation approaches, and cultural expectations regarding care and discharge may limit the generalizability of our findings. Further multicenter prospective studies with larger and more diverse samples are warranted to validate these findings. Third, this study did not include radiological assessment of the degree of spinal cord compression. Lastly, this study focused exclusively on patients with neurologic impairment because of spinal cord compression. However, individuals with metastatic spinal tumors may also experience functional limitations resulting from pain or fatigue without severe motor impairment. Further research is needed to explore predictive factors in this broader patient population.

## Conclusions

This study identified key clinical factors associated with dependence in ADL at discharge among patients with spinal cord dysfunction because of metastatic spinal tumors. Factors such as primary tumor type, severity of motor impairment, systemic inflammation, and persistent pain were independently associated with functional outcomes. These findings highlight the need for early, individualized rehabilitation planning based on oncologic and functional prognostic indicators. Future large-scale, multicenter studies are warranted to confirm these findings and support evidence-based rehabilitation strategies for this growing patient population.

## Supplier

a. JMP Pro, version 18.1; SAS Institute.

## Disclosure

This study was supported by the 10.13039/501100001691Japan Society for the Promotion of Science (JSPS) KAKENHI (Grant Number 23H05371).
